# The magnitude of behavioral isolation is affected by characteristics of the mating community

**DOI:** 10.1002/ece3.1142

**Published:** 2014-06-22

**Authors:** Daniel R Matute

**Affiliations:** 1 Department of Human Genetics, The University of Chicago920 East 58th Street, Chicago, Illinois, 60637; 2 Biology Department, University of North CarolinaChapel Hill, North Carolina, 27599

**Keywords:** Behavioral isolation, *Drosophila*, hybridization

## Abstract

Gene exchange between species occurs in areas of secondary contact, where two species have the opportunity to hybridize. If heterospecific males are more common than conspecific males, females will experience more encounters with males of other species. These encounters might increase the likelihood of heterospecific matings, and lead to the production of hybrid progeny. I studied the mating behavior of two pairs of sibling species endemic to Africa: *Drosophila yakuba/Drosophila santomea* and *Drosophila simulans*/*Drosophila sechellia*. *Drosophila yakuba* and *D. simulans* are cosmopolitan species widely distributed in the African continent, while *D. santomea* and *D. sechellia* are island endemics. These pairs of species hybridize in nature and have the potential to exchange genes in natural conditions. I used these two pairs of *Drosophila* species, and constructed mating communities of different size and different heterospecific:conspecific composition. I found that both the total number of potential mates and the relative frequency of conspecific versus heterospecific males affect female mating decisions in the cosmopolitan species but not in the island endemics. These results suggest that the population characteristics, in which mating occurs, may affect the magnitude of premating isolation. Community composition might thus facilitate, or impair, gene flow between species.

## Introduction

When different animal species come together in the same geographical location and share at least part of their habitat, biological traits associated with mate choice can prevent interbreeding (Coyne and Orr 2004; Price 2007). One of the most effective mechanisms of reproductive isolation results from mate preferences that differ between species (Mayr 1942; Kirkpatrick and Ravigné 2002; Coyne and Orr 2004; Ritchie 2007). Premating behavioral isolation occurs when one or both partners discriminate against the other, thus precluding mating and gene flow (Kaneshiro 1980; Safran et al. 2013). Because of their greater investments of resources during and after mating, females usually are the ones that exert the choice and discriminate against heterospecific males. Mate choice can vary in response to factors intrinsic to the potential partners (i.e., age, health, reproductive fitness), or to extrinsic factors in the environment in which potential mates encounter each other (i.e., habitat and phenology differences; Rice and Hostert 1993; Rolán-Alvarez and Caballero 2000; Knowles et al. 2004; Coyne et al. 2005).

When heterospecific matings occur, and there is not complete intrinsic reproductive isolation between the species, interspecific hybrids are produced. The consequences of hybridization can vary. One potential outcome is that the production of unfit hybrids might impose selection to make premating isolation stronger. In this process, termed reinforcement, the enhancement of premating isolation occurs as a byproduct of selection against maladaptive hybridization (reviewed in Servedio and Noor 2003). A second outcome is that gene flow between species might lead to introgression of foreign genes into the genome; gene flow can have deleterious consequences (e.g., Fang et al. 2012), but also may become raw material for the origin of new adaptations (reviewed in Hedrick 2013). Finally, in rare instances, the production of hybrids can lead to the origin of a new lineage that shows reproductive isolation toward the parentals and thus constitutes a new hybrid species (Arnold and references therein, Schumer et al. 2014).

Even though there is consensus about the evolutionary importance of hybridization in nature, there is not a comprehensive understanding of what biotic or abiotic factors might facilitate hybridization between species. Several arguments posit that if heterospecific males are much more common than conspecific males, then females will experience more encounters with heterospecifics, and produce more hybrid progeny than females in areas where conspecifics are common and heterospecifics are rare (Volpe 1955; Waage 1979; Peterson et al. 2005; Nosil 2012). Nonetheless, the assumption that differential relative frequency of conspecific and heterospecific males can lead to different levels of hybridization has remained untested. Here, I used two pairs of *Drosophila* species to assess whether different ratios of conspecifics to heterospecifics can lead to increased hybridization.

*Drosophila yakuba* and *D. santomea* are two sister species of the *melanogaster* subgroup of species (Lachaise et al. 2000). *Drosophila yakuba* is a human-commensal species that is widespread throughout sub-Saharan Africa (Burla 1954, Lachaise et al. 1988). *Drosophila santomea*, the sister species of *D. yakuba*, is an endemic species to the highlands of São Tomé, a volcanic island off the coast of Cameroon (Lachaise et al. 2000). On Pico de São Tomé, *D. yakuba* inhabits low elevations (below 1450 m), and is found in open habitats (Llopart et al. 2005a). In contrast, *D. santomea* inhabits the mist forests of the island at elevations between 1153 m and 1800 m (Llopart et al. 2005a, Llopart et al. 2005b). Even though these two species diverged around million years ago, they occasionally hybridize in the midlands of the mountain Pico de São Tomé in a recent area of secondary contact (Llopart et al. 2005a, Llopart et al. 2005b). Hybrids between these two species show reduced fitness as a result of intrinsic postzygotic isolation: hybrids of both sexes show mildly reduced viability when compared to pure species. Hybrid females are usually fertile but *F*_1_ hybrid males, and a large proportion of the males from advanced intercrosses, are sterile and show gross defects in spermatogenesis (Moehring et al. 2006, Matute et al. 2009; Matute and Coyne 2010).

*Drosophila simulans* is a human commensal that is thought to have originated in Madagascar and currently has a cosmopolitan geographic range, including the Seychelles archipelago in the Indian Ocean. *Drosophila sechellia* (Tsacas and Bächli 1981; David et al. 2007), an endemic species to the Seychelles, is closely related to *D. simulans* (Amlou et al. 1998; Kliman et al. 2000; McDermott and Kliman 2008). The two species diverged approximately 0.2 million years ago (Kliman et al. 2000; McDermott and Kliman 2008). When the two species are crossed, they produce fertile hybrid females but sterile *F*_1_ males (Coyne and Kreitman 1986; Coyne et al. 1991; Cabot et al. 1994; Hollocher and Wu 1996). In a similar manner to the *D. yakuba/D. santomea* species pair, the majority of males from advanced intercrosses are sterile as well (Moehring et al. 2005). *Drosophila simulans* and *D. sechellia* currently hybridize in the central islands of the Seychelles archipelago (Matute and Ayroles 2014) and are thought to have experienced gene flow at some point (Solignac and Monnerot 1986; Garrigan et al. 2012), indicating that hybridization has played a role in their evolutionary history.

In both species pairs, and both directions of the cross, heterospecific matings occur at a much lower frequency than conspecific matings. In nonchoice experiments (i.e., one female and one male in a vial), females from both *D. yakuba*/*D. santomea* and *D. simulans/D. sechellia* species pairs show a strong tendency to mate with conspecific males and rarely accept heterospecific males (Coyne 1992; Coyne et al. 2002).

In *Drosophila*, attributes of individuals and their environments may affect the frequency of hybridization but they remain largely unstudied in experimental settings. Coyne et al. (2005) explored the effects of three factors – individual female health, the effect of female having more space to escape from unwanted mates, and the effect of having a fruit present in the mating arena – and found that none of them had an effect on female mating decisions. Many other attributes of the mating community remain to be tested.

Population composition may affect female mate choice and the frequency at which hybridization occurs. Here I show that the two population factors, the relative ratio of heterospecific versus conspecific males, and the total number of potential mates, affect female mating decisions in the aforementioned *Drosophila* species pairs. Gene flow between species may therefore be contingent upon the communities in which members of those species encounter one another.

## Methods

### Stocks

All collected stocks and populations were reared on standard cornmeal/Karo/agar medium at 24°C under a 12-h light/dark cycle. I used genetically heterogeneous strains of each species (i.e., synthetic lines) by combining virgin males and females from several isofemale lines collected in São Tomé outside the hybrid zone (i.e., allopatric lines). For all the experiments involving *D. yakuba* and *D. santomea*, I used the *D. yakuba* SYN2005 and *D. santomea* SYN2005 stocks, respectively (Matute et al. 2009; Matute and Coyne 2010). For the experiments involving *D. simulans* and *D. sechellia*, I used *D. simulans* Florida city (Coyne and Beecharn 1987) and *D. sechellia* SYNDenis (Matute and Ayroles 2014) synthetic stocks. All stocks were kept in large numbers after they were created.

### Effect of relative frequency on mating behavior

I explored two components of mating behavior, conspecific copulation latency (i.e., how long does it take for a conspecific mating to take place) and conspecific copulation duration (i.e., how long did copulation last) when *Drosophila* females of four different species (*D. yakuba*, *D. santomea*, *D. simulans*, and *D. sechellia*) were exposed to mating situations in which conspecific males were present and males from a second hybridizing species were also present. I assayed all the possible combinations between the two factors: the relative frequency of heterospecific males (10 different frequencies from 10% to 99% in increments of 10%) and the total number of flies in the mating assay (10 different totals from 100 to 1000 in increments of 100). In total, I recorded both premating isolation behaviors in 100 combinations and assayed 15 females per combination.

All measurements of premating isolation were carried out using previously described methods (Coyne et al. 2002; Matute 2010); unlike previous experiments, females were allowed to choose their mate. Briefly, females and males were collected as virgins within 8 h of eclosion. All individuals were kept in vials of 22 flies of the same sex for 3 days. On Day 3, females were housed in red-colored food (which turned their abdomens red) for their easier identification in the experimental mating population. Red dye has a negligible effect on mating choice (Ting et al. 2001; Matute 2013). Four days after collection, a single virgin female and the virgin males dictated by each experimental combination, as described above, were combined. All flies were transferred without anesthesia to a bottle with cornmeal food. For each mated female, I recorded conspecific copulation latency (i.e., how long did it take for a *D. yakuba* female to find a conspecific mating partner and start copulating), and conspecific copulation duration (i.e., how long did conspecific females and males remained joint in mating). Flies were observed for 1 h. I did not observe any heterospecific matings using this approach.

In parallel, I set up mating trails in which the only males present were conspecific. I varied the number of flies in the mating assay (10 different totals from 100 to 1000 in increments of 100). I recorded both conspecific copulation latency and conspecific copulation duration in mating trials of ten different sizes and assayed 15 females per population size.

To study heterospecific mating frequency (i.e., how often females accepted heterospecific males even though conspecific males were present in the population), I measured premating isolation over 24 h for a single female that were housed with both conspecific and heterospecific males in the numbers and relative frequencies described above. I let trials proceed for 24 h and then anesthetized all the flies in the mating population with CO_2_, discarded the males and returned each female to the vial where the mating took place in order to observe the resulting *F*_1_ generation. I started 100 replicates per combination of variables (i.e., each combination of heterospecific relative frequency and total number of flies). I raised the progeny of each female by standard fly husbandry methods, and used the presence or absence of hybrid progeny as a conservative proxy of whether heterospecific matings occurred. The frequency of heterospecific matings for each treatment was calculated by counting how many of the vials within a block produced hybrid progeny. For *D. yakuba* and *D. santomea* experimental populations, I used abdominal pigmentation to identify whether vials produced hybrid progeny. I qualitatively scored the abdominal pigmentation of 100 males per vial. *Drosophila yakuba* males have a dark abdomen, while *D. santomea* males have a light abdomen. *F*_1_ males have an intermediate abdominal pigmentation (Llopart et al. 2002). For *D. simulans* and *D. sechellia*, experimental populations, I used male genital morphology to identify whether vials produced hybrid progeny. I qualitatively scored the morphology of the posterior genital arch of 100 males per vial. *Drosophila simulans* males have red spheroid large genital arches. *Drosophila sechellia* males have small and elongated genital arches. The hybrids show intermediate genital morphology and can be distinguished from the parental species (Coyne and Kreitman 1986; Coyne et al. 1991; MacDonald and Goldstein 1999; Matute and Ayroles 2014).

### Statistical analyses

All statistical analyses were carried out using R (R Development Core Team 2005).

To determine the significance of the frequency of heterospecific males and the mating population size on mating behavior, I analyzed the conspecific copulation latency and conspecific copulation duration, I fitted a multiple regression for each of the two components of premating behavior. The premating behavior trait was the response, while the relative frequency of heterospecific males and the total number of flies were the continuous variables. Multiple regressions took the form:


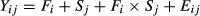


where *Y*_*ij*_ is the response (behavioral trait), *F*_*i*_ is the frequency of heterospecific males, *S*_*j*_ is the mating population size (i.e., number of males in the mating assay), *F*_*i*_ × *S*_*j*_ is the interaction between the variables, and *E*_*ij*_ is the error term. The significance of independent relationships in the multiple regressions was determined using a two-tailed *t*-test on the regression coefficients (degrees of freedom, df = 1496).

For the heterospecific mating frequency data (collected when females were housed with different combinations of males over 24 h), I used the “aod” package in R (Lesnoff and Lancelot 2012). I fitted generalized logistic models with binomial response distributions in which whether a vial produced hybrid progeny or not was the response. The total number of flies in the vial and the relative frequency of heterospecifics were considered continuous variables. I allowed for an interaction term between these two variables. Significance of each variable was assessed using a Wald text following a *χ*^2^ distribution (df = 1). Linear models followed the form:


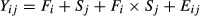


*P* values under 0.05 were considered significant.

I assessed whether total number of flies and the ratio of heterospecifics were multicollinear variables (i.e., the two predictor variables were correlated). The reason for this concern was that the ratio of heterospecifics was calculated using the total number of flies as the denominator. This can lead to autocorrelation of the two variables. I calculated the variance inflation factor (VIF) between the two regression coefficients using the R package HH (Heiberger 2009). The VIF for predictor *i* equals:


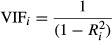


where 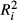 is the coefficient of determination from a regression of predictor *i* against the remaining predictors. In the case of the eight multiple regressions shown in this study, we calculated the VIF values for the two predictor variables in models that had no interaction term. Because all the multiple regressions had the same two predictor variables (and the same values), I only needed to calculate one VIF. The VIF for the model


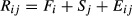


where *R*_*ij*_ is either latency or duration, *F*_*i*_ is the ratio of heterospecifics and *S*_*j*_ is the mating population size, equaled 1. Values of VIF larger than 5 are considered evidence of collinearity between predictor variables (Stine 1995; O'Brien 2007). This result indicates that the two predictor variables are not multicollinear.

To plot heterospecific mating frequency data, I used Akima interpolation splines (Akima 1970) between sampling intervals, and plotted them using contour maps with the “akima” R package (Akima et al. 2006).

## Results

### *D. yakuba*/*D. santomea*

**Figure 1:**
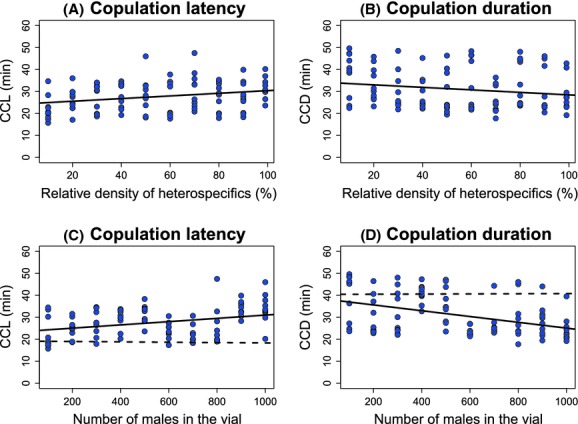
High ratios of *Drosophila santomea* males relative to *Drosophila yakuba* males affect two components of mating sexual behavior (conspecific copulation latency –CCL– and conspecific copulation duration –CCD–) in *D. yakuba* females. (A) The number of *D. santomea* males (%) significantly affected conspecific copulation latency in *D. yakuba* females. (B) The number of *D. santomea* males (%) significantly affected conspecific copulation duration in *D. yakuba* females. (C) The size of the mating population significantly affected conspecific copulation latency in *D. yakuba* females. (D) The size of the mating population significantly affected conspecific copulation duration in *D. yakuba* females. Each circle represents the average of 15 replicates. All significance values are shown in Table 1.

I studied the mating sexual behavior of *D. yakuba* females exposed to different relative frequencies of *D. santomea* and *D. yakuba* males in populations with different total numbers of males. The mating behaviors I studied were conspecific copulation latency (time until mating began) and duration. The average conspecific copulation latency and conspecific copulation duration per treatment are shown in Figure 1. Both the relative frequency of *D. santomea* males and population size affected the mating behavior of *D. yakuba* females toward conspecific males; in larger populations, the conspecific copulation latency was longer (Table 1, Fig. 1) and conspecific copulation duration was shorter (Table 2, Fig. 1). In the case of duration, but not of latency, the interaction between population size and composition was also significant, indicating a strong interplay between the size of the mating community and the ratio of heterospecific to conspecific males (Tables 1, 2).

**Table 1:** Multiple regressions for copulation latency for each of the four species

Copulation latency												
	Intercept			Relative frequency of heterospecifics			Total number of flies			Interaction: Relative frequency of heterospecifics × Total number of flies		
				
	Estimate	*t* value	*P*-value	Coefficient	*t* value	*P*-value	Coefficient	*t* value	*P*-value	Coefficient	*t* value	*P*-value
*Drosophila yakuba*	20.99	16.639	<1 × 10^−10^	**4.69 × 10**^**−2**^	**2.302**	**0.022**	**6.10 × 10**^**−3**^	**3.001**	**2.74 × 10**^**−3**^	2.44 × 10^−5^	0.744	0.457
Drosophila *santomea*	18.75	17.404	<1 × 10^−10^	9.38 × 10^−4^	0.054	0.957	1.39 × 10^−3^	0.802	0.423	−1.03 × 10^−5^	−0.367	0.714
*Drosophila simulans*	27.56	24.868	<1 × 10^−10^	**4.80 × 10**^**−2**^	**2.680**	**7.45 × 10**^**−3**^	**−1.00 × 10**^**−2**^	**−5.608**	**2.43 × 10**^**−8**^	**6.79 × 10**^**−5**^	**2.353**	**0.019**
*Drosophila sechellia*	43.58	33.979	<1 × 10^−10^	2.35 × 10^−2^	1.135	0.259	2.25 × 10^−3^	1.086	0.280	−4.35 × 10^−5^	−1.301	0.196

A multiple linear regression was fitted to study the effect of number of heterospecifics (*F*_*i*_), the population size (*S*_*j*_), and the interaction between these two variables (*F*_*i*_ × *S*_*j*_) in the copulation latency of each of the four species in experimental cages (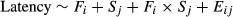). The two factors and the interaction were significant for *D. yakuba* and *D. simulans* but not for *D. santomea* or *D. sechellia*. Significant values (*P* < 0.05, df = 1496) are marked in bold.

**Table 2:** Multiple regressions for copulation duration for each of the four species

Copulation duration												
	Intercept			Relative frequency of heterospecifics			Total number of flies			Interaction: Relative frequency of heterospecifics × Total number of flies		
				
	Estimate	*t* value	*P*-value	Coefficient	*t* value	*P*-value	Coefficient	*t* value	*P*-value	Coefficient	*t* value	*P*-value
*Drosophila yakuba*	45.41	30.847	<1 × 10^−10^	**0.13**	**−5.469**	**5.30 × 10**^**−8**^	**−2.05 × 10**^**−2**^	**−8.625**	**1 × 10**^**−10**^	**1.33 × 10**^**−4**^	**3.458**	**5.58 × 10**^**−4**^
Drosophila *santomea*	46.62	47.808	<1 × 10^−10^	2.07 × 10^−2^	1.312	0.190	2.87 × 10^−4^	0.182	0.855	−2.60 × 10^−5^	−1.022	0.307
*Drosophila simulans*	41.18	27.189	<1 × 10^−10^	**−0.12**	**−4.694**	**2.92 × 10**^**−6**^	**−9.78 × 10**^**−3**^	**−4.005**	**6.50 × 10**^**−5**^	**8.01 × 10**^**−5**^	**2.030**	**0.043**
*Drosophila sechellia*	15.36	19.299	<1 × 10^−10^	8.56 × 10^−4^	0.067	0.947	−5.27 × 10^−4^	−0.411	0.681	−6.26 × 10^−6^	−0.302	0.763

A multiple linear regression was fitted to study the effect of number of heterospecifics (*F*_*i*_), the population size (*S*_*j*_), and the interaction between these two variables (*F*_*i*_ × *S*_*j*_) in the copulation duration of each of the four species in experimental cages (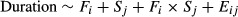). The two factors and the interaction were significant for *D. yakuba* and *D. simulans* but not for *D. santomea* or *D. sechellia*. Significant values (*P* < 0.05 df = 1496) are marked in bold.

Notably when a single *D. yakuba* female was housed with only conspecific males in populations of different sizes, both copulation latency and duration remained constant (Table 3). These results indicate that a simple increase in the population size (and consequently the sex ratio) does not lead to changes in mating behavior. Such changes are only observed when there are heterospecific males present in the assay.

**Table 3:** Mating assays with only conspecific males and different population sizes

	Copulation latency			Copulation duration		
		
Species	Coefficient	*t* value	*P*-value	Coefficient	*t* value	*P*-value
*Drosophila yakuba*	−8.009 × 10^−4^	−0.537	0.592	3.25 × 10^−4^	0.144	0.885
Drosophila *santomea*	4.746 × 10^−4^	0.381	0.704	−4.875 × 10^−4^	−0.318	0.751
*Drosophila simulans*	2.142 × 10^−3^	1.30	0.196	−1.289 × 10^−3^	−0.734	0.464
*Drosophila sechellia*	9.093 × 10^−4^	0.758	0.449	−1.776 × 10^−3^	−1.201	0.232

No heterogeneity was detected in any of the four species at the two mating behaviors. I found no significant values (*P* < 0.05, df = 1496).

**Figure 2:**
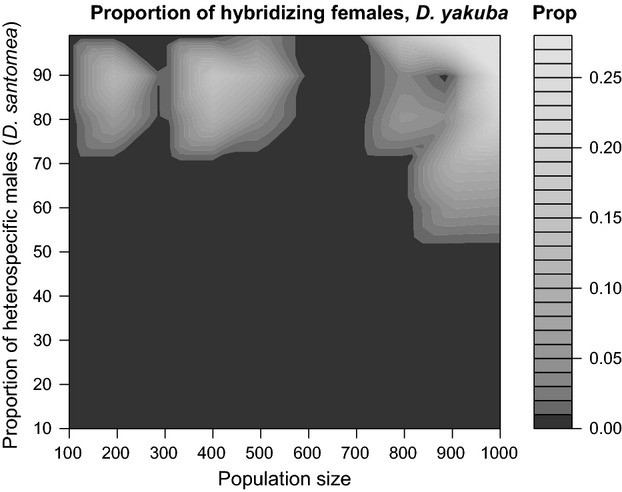
The ratio of *Drosophila santomea* males relative to that of *Drosophila yakuba* males affects the strength of premating isolation in *D. yakuba* females. In conditions where *D. santomea* males are overwhelmingly more common than *D. yakuba* males, *D. yakuba* females accept *D. santomea* males. Light gray indicates conditions where heterospecific matings were observed.

In addition to conspecific copulation latency and duration, I investigated the effect of community composition on reproductive isolation in *D. yakuba*. There were no heterospecific matings observed in the 1-h trials, so I next looked at heterospecific mating frequencies in 24-h trials when *D. yakuba* females were housed with the same ratios of conspecific:heterospecific males and total numbers of males as in the 1-h observations. In this experiment, large populations that had a high ratio of heterospecific to conspecific males were more prone to produce hybrid progeny (binomial logistic regression, Wald test, *χ*^2^ = 384.9, df = 3, *P* < 1 × 10^−10^, Fig. 2). I found that the ratio of heterospecific to conspecific males had a significant effect on the frequency of heterospecific matings (Wald test, *χ*^2^ = 59.1, df = 1, *P* < 1.1 × 10^−10^). The total number of flies also had a significant effect on the frequency of heterospecific matings (*χ*^2^ = 35.4, df = 1, *P* = 2.9 × 10^−9^). The interaction between these two variables was also significant (*χ*^2^ = 11.9, df = 1, *P* = 5.60 × 10^−4^). In general, heterospecific matings were observed only in populations in which more than 70% of the males were *D. santomea*.

**Figure 3:**
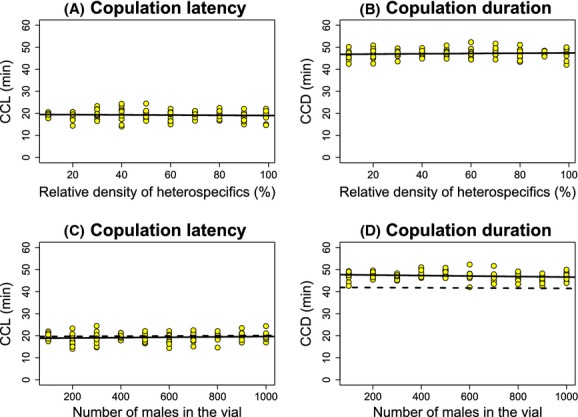
The ratio of *Drosophila yakuba* males relative to *Drosophila santomea* males had no effect on two components of mating sexual behavior (conspecific copulation latency –CCL– and conspecific copulation duration –CCD–) in *D. santomea* females. (A) The number of *D. yakuba* males (%) did not affect conspecific copulation latency in *D. santomea* females. (B) The number of *D. yakuba* males (%) did not affect conspecific copulation duration in *Drosophila santomea* females. (C) The size of the mating population did not affect conspecific copulation latency in *D. santomea* females. (D) The size of the mating population did not affect conspecific copulation duration in *D. santomea* females. Each circle represents the average of 15 replicates. All significance values are shown in Table 1.

I next looked at the mating behavior of females from the other species of this pair. Using the same procedures but with the species reversed, I investigated whether conspecific mating behavior or levels of reproductive isolation in *D. santomea* females were also affected by the ratio of heterospecific versus conspecific males and the size of the mating population. Neither of the two factors that I manipulated (as above, ratio of heterospecific to conspecific males and the mating population size) led to changes of the magnitude of reproductive isolation in *D. santomea* females toward *D. yakuba* males, nor to changes in the conspecific copulation latency (Table 1) or duration of *D. santomea* females (Table 2). In controls that only included different numbers of *D. santomea* males I observed no differences in copulation latency or copulation duration among treatments (Fig. 3, Table 3). These results indicate that the mating behavior of *D. santomea* females is not affected by the presence of heterospecific males regardless of the frequency at which they are present. Furthermore, in populations where both *D. yakuba* and *D. santomea* males were present, I observed no heterospecific copulations, neither in the 1-h measurements (*n* = 1500 observations and 100 different treatments) nor in the 24-h trials (*n* = 10,000 observations and 100 different treatments). Although they will occasionally mate with *D. yakuba* males in no-choice matings (Coyne et al. 2002), *D. santomea* females will always choose *D. santomea* males when given the chance.

### D. simulans/D. sechellia

**Figure 4:**
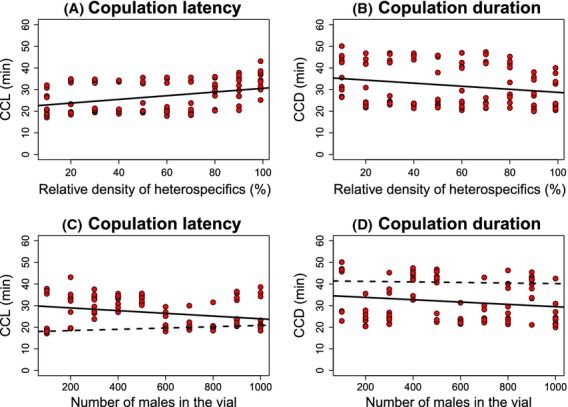
High ratios of *Drosophila sechellia* males relative to *Drosophila simulans* males affect two components of mating sexual behavior (conspecific copulation latency –CCL– and conspecific copulation duration –CCD–) in *D. simulans* females. (A) The number of *D. sechellia* males (%) significantly affected conspecific copulation latency in *D. simulans* females. (B) The number of *D. sechellia* males (%) significantly affected conspecific copulation duration in *D. simulans* females. (C) The size of the mating population significantly affected conspecific copulation latency in *D. simulans* females. (D) The size of the mating population significantly affected conspecific copulation duration in *D. simulans* females. Each circle represents the average of 15 replicates. All statistics and significance values are shown in Table 1.

In order to assess whether the results observed for *D. yakuba/D. santomea* were specific to that species pair or whether they also applied to other species; I followed the same experimental procedures in a second species pair: *D. simulans* and *D. sechellia*. I measured the magnitude of the two conspecific mating behavior traits in *D. simulans* females exposed to different ratios of *D. sechellia* to *D. simulans* males. For 1-h experiments, multiple regressions showed that the frequency of the heterospecific *D. sechellia,* and the total number of flies affected both components of mating behavior in each mating trial for *D. simulans* females (see Methods, Table 1 and 2, Fig. 4). The interaction between these two variables was marginally significant for conspecific latency (Table 1) and conspecific duration (Table 2). I also assayed mating behavior in cages that contained only *D. simulans* males and observed no variation in latency or duration (Table 3). These results, similar to the ones from *D. yakuba*, suggest that a simple increase in population size is not enough to modify the mating behavior of *D. simulans* females and that changes in mating behavior only occur when there are heterospecific males present in the assay (Table 3, Fig. 4).

**Figure 5:**
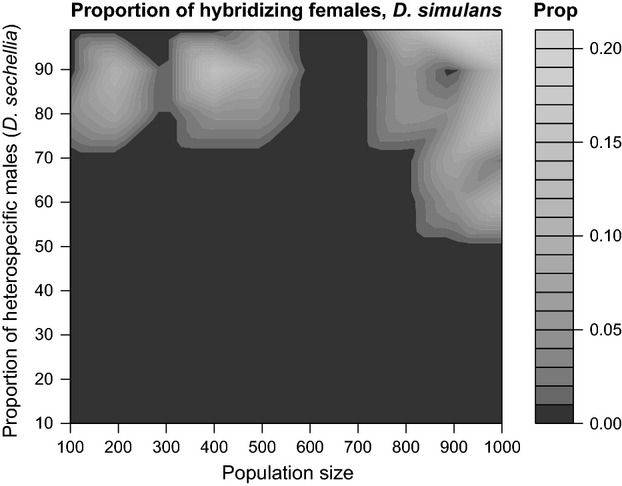
The ratio of *Drosophila sechellia* males relative to that of *Drosophila simulans* males affects the strength of premating isolation in *D. simulans* females. In conditions where *Drosophila sechellia* males are overwhelmingly more common than *D. simulans* males, *D. simulans* females accept *D. sechellia* males.

While both latency and duration of conspecific matings were affected by the presence and number of heterospecifics, no heterospecific matings were observed in these 1-h trials either. Next I examined heterospecific matings when I housed *D. simulans* females with different combinations of *D. simulans* and *D. sechellia* males for 24 h. Similar to the observations of *D. yakuba*, large populations with few conspecific males were more likely to produce hybrid progeny (binomial logistic regression, Wald test, *χ*^2^ = 361.1, df = 3, *P* < 1.4 × 10^−13^, Fig. 5). I found there was a strong effect on the number of heterospecific matings of the relative frequency of conspecific to heterospecific males (*χ*^2^ = 54.7, df = 1, *P* = 1.9 × 10^−6^), the total number of flies in the cage (*χ*^2^ = 22.7, df = 1, *P* = 1.9 × 10^−6^), and the interaction between these two variables (*χ*^2^ = 6.20, df = 1, *P* = 0.013).

**Figure 6:**
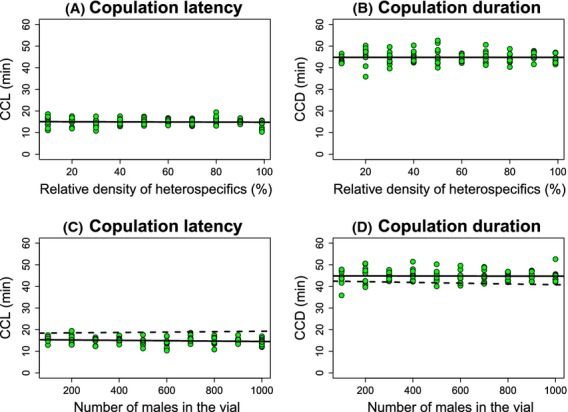
The ratio of *Drosophila simulans* males relative to *Drosophila sechellia* males had no effect on two components of premating sexual behavior (conspecific copulation latency –CCL– and conspecific copulation duration –CCD–) in *D. sechellia* females. (A) The number of *D. simulans* males (%) did not affect conspecific copulation latency in *D. sechellia* females. (B) The number of *D. simulans* males (%) did not affect conspecific copulation duration in *D. sechellia* females. (C) The size of the mating population did not affect conspecific copulation latency in *D. sechellia* females. (D) The size of the mating population did not affect conspecific copulation duration in *D. sechellia* females. Each circle represents the average of 15 replicates. All statistics and significance values are shown in Table 1.

Finally, I studied the mating behavior of females from the second species of this pair, *D. sechellia*, in communities of different sizes and compositions. Neither of the two experimental variables (population size nor relative frequency of heterospecifics) affected the mating behavior (conspecific mating latency or duration) of *D. sechellia* females (Tables 1, 2, Fig. 6). I observed no changes in copulation latency or copulation duration when the assays only included *D. sechellia* males (Table 3).

No heterospecific matings were observed with *D. sechellia* for either 1-h or 24-h trials. These results indicate that, as was the case for *D. santomea*, the mating behavior of *D. sechellia* females is not affected by the presence of heterospecific males regardless of the ratio of heterospecific to conspecific males and the mating population size.

## Discussion

This study explores how relative frequency and population size, individually and interacting, influence mating behavior in at least two species of *Drosophila*. The results shown here have two implications for our understanding of how premating isolation is involved in the persistence of potentially hybridizing species. First, they confirm that, in some animal species, hybridization is more likely if heterospecific males are disproportionally more abundant than conspecifics males. The relative frequency of males has been proposed as a factor influencing the magnitude of reproductive isolation in natural populations (Harper and Paulson 1994; Berglund 1995; Sprenger et al. 2011; Willis et al. 2011, 2012; Verzijden et al. 2012). Under this regime, females will accept males from other species if the environment in which mating takes place is not conducive to finding a conspecific partner – for instance, if females cannot detect the few conspecific males in the community, or if they encounter many heterospecific males before a conspecific (Wilson and Hedrick 1982; Willis et al. 2012). These results are of particular importance for the study of reinforcing selection, the enhancement of prezygotic isolation as a byproduct of maladaptive hybridization (reviewed in Servedio and Noor 2003; Coyne and Orr 2004; Pfennig and Pfennig 2009). It is commonly argued that if reinforcing selection affects only one species in a hybrid zone, it should be the rarest one (Coyne and Orr 2004; Price 2007; Nosil 2012). The basic premise of this assertion is that the less abundant species will be more at risk of hybridizing because they will face more encounters with heterospecific mates. This claim makes intuitive sense but has remained largely untested. My results provide strong evidence that for some *Drosophila* species, the less abundant species in a community is more at risk of mating heterospecifically.

Not only are *D. yakuba* and *D. simulans* less abundant in their respective hybrid zones, but they are more widespread outside of the hybrid zone. *Drosophila santomea* and *D. sechellia*, however, are endemic to their island habitats. Behavioral isolation is much stronger when the matings involve females from the island endemics than when they involve females from the widespread species, regardless of the experimental design used to quantify the reproductive isolation (Coyne 1992; Coyne et al. 2002). The results from this report are in the same vein of previous studies and show that even in environments in which heterospecific males are overwhelmingly more common than conspecific males, *D. santomea* and *D. sechellia* females strongly discriminate against heterospecific males (Coyne 1996; Tomaru et al. 2004).

Finally, I also demonstrate that the size of the mating population has an effect on the likelihood of the occurrence of heterospecific matings. Because each assay was composed with a single female, when the population size of the male conspecific varies, the sex ratio also varies. This means I cannot fully disentangle the effects of population size and sex ratio with the current set of data, and direct observations of multifemale populations would pose distinct challenges. The results from the mating assays with only conspecific males do allow me to rule out that a simple increase in the population size (and consequently the sex ratio) causes changes in copulation latency and copulation duration. Instead, changes in these behaviors are only observed when heterospecific males are present in the assay. Regardless, these results indicate that the characteristics of the community in which mating takes place can affect the magnitude of reproductive isolation between potentially hybridizing species.

The effects of biotic factors on hybridization are largely unexplored in animals but not in plants. Pollen dispersal, a major factor leading to gene flow in plants, is heavily influenced by heterospecific relative frequency in nature (Campbell and Halama 1993; Bosch and Waser 1999, 2001). Studies on pollinator competition have revealed that the presence of heterospecifics at different densities can affect the relative rates of interspecific pollen transfer (Price and Waser 1982; Kohn and Waser 1985; Campbell and Waser 1989; Campbell and Halama 1993; Bosch and Waser 2001; reviewed in Mitchell et al. 2009). The nature of reproductive isolation differs dramatically between plants and animals with internal fertilization; while the results in plants are pollinator-mediated and thus extrinsic, the results shown here are intrinsic to the decision-making process of females choosing whether to accept a potential mate.

Even though there is little empirical evidence for potential effects of the relative frequency of heterospecific to conspecific males on the magnitude of reproductive isolation in animals, there is no reason why heterospecific males cannot be seen as low-quality males and conspecifics as high-quality males (Penteriani 2003; Kokko and Rankin 2006). This is bound to be especially true in those cases in which postzygotic isolation is already in place, as happens in the two studied species pairs (i.e., hybrid males from the crosses *D. yakuba* × *D. santomea*, and from the crosses *D. simulans* × *D. sechellia* are completely sterile). Many mating systems have demonstrated that when males interact in nature, high-quality males usually win the competition for females (Howard et al. 1997, 1998; Correa and Thiel 2003; Thiel and Correa 2004). Under some circumstances, however, females may not be able to exert their preferences (Hirotani 1994). In dung flies and colonial blackbirds, females are only able to realize their preferences for high-quality males in low-density populations. High-quality males cannot exclude other males and low-quality males get access to females when population density is high (Borgia 1981; Webster and Robinson 1999; Wong and Candolin 2005). The results here shown indicate that high densities of heterospecific males (low quality) interfere with the decision process that *Drosophila* females must make in order to choose conspecific over heterospecific males.

This report demonstrates that at least for these two pairs of species of *Drosophila*, a high relative frequency of heterospecific males and a large mating population size can affect mating behaviors and lead to increased levels of hybridization even in situations in which females have access to conspecific males. This might recapitulate the situation at the edges of hybrid zones where the relative frequency of one of the species is low; in these cases, females might be exposed to high ratios of heterospecific to conspecific males, which in turn might lead to interspecific matings and increase the likelihood of admixture and gene flow.

## Supporting Information

**Data S1.** Raw data and analytical code used in this report. Dryad link: doi: 10.5061/dryad.fp861.
